# The Occult Powers

**Published:** 1890-08

**Authors:** 


### THE OCCULT POWERS.

This is an age of progress in all things relating to physics, and the subjection of the plainly observable forces of nature to the requirements of man, in his most advanced civilization.

The inventive faculties have been given full play, and new and important discoveries made in untried fields hitherto looked upon as forbidden ground. The road to scientific achievement has been broadened into an open highway, wherein the humblest votary may pursue his onward way without the fear of scholastic denial or ecclesiastic censure. It is only in the occult region of inquiry that a determined resistance is met, for there is nothing quite so difficult to overcome as the fixed opinions of the unlearned and self-satisfied. Their treatment of a question is almost wholly from a materialistic point of view. Any thing beyond the reach of their physical senses is apt to be ignored by them as speculative or impossible, hence unworthy of serious investigation. The superabundant proofs of entities and forces, undreamt of in their philosophy, are tossed aside as the vagaries of a visionary.

There has been put forth in the city of Boston once a week, for more than thirty years, a publication devoted to the promulgation of occult truth. It is known as *The Banner of Light.* Its editors are earnest men of ample scholarship and large experience in the chosen field of their ill-requited labors. Each issue of this paper contains a number of communications purporting to have been given by denizens of the super-mundane world, who lived out their lives here, as we are living out ours now. They give facts in detail, to establish their identity, name, place of earthly residence, period of earth-life, and date and cause of their so-called death. Many, very many of these messages have been vouched for as true, even to the minutest particular, and so far as we are informed, not one of the published thousands has been shown to be false. Is there nothing in this startling fact which should awaken an honest inquiry into it from the very centres of intelligence? Yet the *Banner* is only one of many others of the same class. If it is the demonstration of a *truth*, all men and orders and creeds should know it, and take it home to their hearts and their daily lives. If, on the other hand, it is *false*, it ought to be so thoroughly exposed as never to repeat its abominations. It is only falsehood, that is to be feared. Truth should never be eluded or obscured, lest it disturb settled views, as it is much too often. For centuries the world was held in darkness by the worst of errors ecclesiastically enjoined and cruelly enforced. By all means, let us know all that is capable of being known of this bright and beautiful realm of the occult.

We are glad to be able to state that more recently the frequency of these phenomena have drawn to them the attention of some of the wisest and ablest minds of the two hemispheres, till it is no longer a thing to be wondered at, to find the laboratory of the scientist given to their scrutiny, and the pulpit here and there bold enough to do justice to a new idea and open the way to a glimmering of the new light, but

"Not to the multitude, oh! not to them,
But to the sacred few, the circle small,
Which found thy world and was thy all in all."

